# Supplementary material for: Mechanisms governing the pioneering and redistribution capabilities of the non-classical pioneer PU.1
Source: Nat Commun. 2020 Jan 21;11:402. doi: 10.1038/s41467-019-13960-2 (PMC6972792; doi:10.1038/s41467-019-13960-2)
Supplement: Supplementary file 7 — Source data [file 41467_2019_13960_MOESM7_ESM.zip › Source_Data/FigureS2/FigureS2B_MotifScanOutput/KmeansCluster13/homerResults.html]

/misc/data/analysis/project\_PU1/CTV1/motifs/basic/PU1\_Kmeans14.13/final - Homer de novo Motif Results


# Homer *de novo* Motif Results (/misc/data/analysis/project\_PU1/CTV1/motifs/basic/PU1\_Kmeans14.13/final)

Known Motif Enrichment Results  
Gene Ontology Enrichment Results  
If Homer is having trouble matching a motif to a known motif, try copy/pasting the matrix file into
STAMP  
More information on motif finding results: HOMER
| Description of Results
| Tips
  
Total target sequences = 1692  
Total background sequences = 45096  
\* - possible false positive  

|  |  |  |  |  |  |  |  |  |
| --- | --- | --- | --- | --- | --- | --- | --- | --- |
| Rank | Motif | P-value | log P-pvalue | % of Targets | % of Background | STD(Bg STD) | Best Match/Details | Motif File |
| 1 | T C A G C T G A T A C G T G C A A C T G A C T G C G T A C G T A T C A G A G C T T A C G T C A G | 1e-608 | -1.402e+03 | 73.40% | 14.61% | 37.6bp (69.7bp) | ETS1(ETS)/Jurkat-ETS1-ChIP-Seq(GSE17954)/Homer(0.954) More Information \| Similar Motifs Found | motif file (matrix) |
| 2 | T A C G T C A G C G T A C T G A T C G A A G T C A G T C C G T A G T A C G T C A | 1e-127 | -2.935e+02 | 32.39% | 10.27% | 53.1bp (62.8bp) | RUNX1(Runt)/Jurkat-RUNX1-ChIP-Seq(GSE29180)/Homer(0.977) More Information \| Similar Motifs Found | motif file (matrix) |
| 3 | C T A G C T A G A T C G T G A C C T A G A T C G T C G A T C G A T A G C G A T C G C T A | 1e-65 | -1.507e+02 | 31.74% | 14.78% | 53.2bp (67.6bp) | POL011.1\_XCPE1/Jaspar(0.849) More Information \| Similar Motifs Found | motif file (matrix) |
| 4 | T C G A T A G C A G C T A C G T T A G C G A T C C A G T T C A G A G T C G C A T A G C T | 1e-48 | -1.113e+02 | 7.98% | 1.55% | 53.7bp (67.0bp) | Foxo1/MA0480.1/Jaspar(0.698) More Information \| Similar Motifs Found | motif file (matrix) |
| 5 | G T C A A T G C G T A C A T C G C T A G C G T A C T G A G T C A A G C T | 1e-46 | -1.076e+02 | 9.34% | 2.21% | 53.0bp (70.6bp) | ETV2/MA0762.1/Jaspar(0.931) More Information \| Similar Motifs Found | motif file (matrix) |
| 6 | T A G C C G A T C A G T G T A C G A T C C A T G G A T C C G A T C A G T | 1e-43 | -9.952e+01 | 50.53% | 33.79% | 57.1bp (68.8bp) | POL010.1\_DCE\_S\_III/Jaspar(0.706) More Information \| Similar Motifs Found | motif file (matrix) |
| 7 | T G A C G C T A A T C G A G C T A G C T G T A C A G T C A G C T A G T C G T A C G A T C G T C A A G T C G C T A | 1e-40 | -9.229e+01 | 8.69% | 2.22% | 45.0bp (64.1bp) | Sp5(Zf)/mES-Sp5.Flag-ChIP-Seq(GSE72989)/Homer(0.679) More Information \| Similar Motifs Found | motif file (matrix) |
| 8 | G C A T C G A T A T G C A C G T A C G T T G A C A G T C G A C T C T A G C G A T A G T C C G A T | 1e-38 | -8.886e+01 | 5.56% | 0.92% | 56.0bp (69.4bp) | Ets1-distal(ETS)/CD4+-PolII-ChIP-Seq(Barski\_et\_al.)/Homer(0.823) More Information \| Similar Motifs Found | motif file (matrix) |
| 9 | A T G C T C G A A T G C G C T A A G C T A G T C G A T C C G A T G T A C G C T A C T A G | 1e-36 | -8.485e+01 | 5.79% | 1.06% | 54.4bp (66.5bp) | PU.1(ETS)/ThioMac-PU.1-ChIP-Seq(GSE21512)/Homer(0.769) More Information \| Similar Motifs Found | motif file (matrix) |
| 10 | G T A C A G T C G C A T C G T A A C G T A G T C C T G A T C A G | 1e-35 | -8.128e+01 | 20.27% | 9.85% | 57.8bp (64.6bp) | GLN3/MA0307.1/Jaspar(0.910) More Information \| Similar Motifs Found | motif file (matrix) |
| 11 | A T C G C G A T A C T G A T C G A G C T A G C T A C G T A C T G C A G T A C T G A T C G A C G T | 1e-33 | -7.707e+01 | 4.37% | 0.64% | 55.1bp (58.4bp) | RUNX2(Runt)/PCa-RUNX2-ChIP-Seq(GSE33889)/Homer(0.809) More Information \| Similar Motifs Found | motif file (matrix) |
| 12 | A C G T T A C G C G T A A T C G A C G T A T G C C G T A A C T G G T A C G A T C T G C A | 1e-32 | -7.427e+01 | 8.33% | 2.46% | 52.0bp (62.4bp) | JunB(bZIP)/DendriticCells-Junb-ChIP-Seq(GSE36099)/Homer(0.964) More Information \| Similar Motifs Found | motif file (matrix) |
| 13 | C T A G C A G T C T A G T C A G C T A G A G T C A C T G A C T G C A G T | 1e-31 | -7.249e+01 | 23.58% | 12.84% | 56.8bp (68.6bp) | btd/dmmpmm(Noyes)/fly(0.889) More Information \| Similar Motifs Found | motif file (matrix) |
| 14 | G C A T C A T G G T A C A C T G A C G T G T A C C G T A | 1e-30 | -7.019e+01 | 11.76% | 4.59% | 53.0bp (64.8bp) | RTG3(MacIsaac)/Yeast(0.909) More Information \| Similar Motifs Found | motif file (matrix) |
| 15 | A G T C A T G C C G T A A G T C C G T A A C G T A G T C | 1e-28 | -6.624e+01 | 10.11% | 3.71% | 52.8bp (65.6bp) | ETS:RUNX(ETS,Runt)/Jurkat-RUNX1-ChIP-Seq(GSE17954)/Homer(0.829) More Information \| Similar Motifs Found | motif file (matrix) |
| 16 | G T C A A G T C A G C T A G C T A G T C A G T C A C G T G T A C G C A T A C G T A G T C A G T C G C A T | 1e-28 | -6.483e+01 | 5.20% | 1.14% | 63.8bp (68.8bp) | PU.1(ETS)/ThioMac-PU.1-ChIP-Seq(GSE21512)/Homer(0.764) More Information \| Similar Motifs Found | motif file (matrix) |
| 17 | T C G A A T G C C G T A A C G T G T A C C G T A T G C A C T G A C T A G T A G C | 1e-27 | -6.317e+01 | 13.36% | 5.92% | 53.4bp (63.6bp) | Tcf3(HMG)/mES-Tcf3-ChIP-Seq(GSE11724)/Homer(0.927) More Information \| Similar Motifs Found | motif file (matrix) |
| 18 | A T G C T A G C C T A G T A C G C T G A T G C A G T A C A G T C C A T G T A C G T G C A T C G A | 1e-27 | -6.230e+01 | 4.61% | 0.93% | 58.9bp (75.8bp) | LEU3/LEU3\_SM/47-LEU3(Harbison)/Yeast(0.805) More Information \| Similar Motifs Found | motif file (matrix) |
| 19 | A T G C G A C T C A G T T C G A C A G T G T A C C G T A T A C G G T C A C G T A | 1e-25 | -5.813e+01 | 9.46% | 3.61% | 58.1bp (63.7bp) | PB0022.1\_Gata5\_1/Jaspar(0.853) More Information \| Similar Motifs Found | motif file (matrix) |
| 20 | A T G C A G T C T C G A C T A G A G T C A G T C C G T A C G T A A G C T A T G C T C G A | 1e-25 | -5.809e+01 | 6.80% | 2.08% | 58.3bp (63.4bp) | POL004.1\_CCAAT-box/Jaspar(0.945) More Information \| Similar Motifs Found | motif file (matrix) |
| 21 | C T G A C T G A C T G A T C G A T C A G C T A G T C A G C T G A T C G A T A G C G C A T | 1e-23 | -5.429e+01 | 12.35% | 5.67% | 48.4bp (68.6bp) | SpiB(ETS)/OCILY3-SPIB-ChIP-Seq(GSE56857)/Homer(0.777) More Information \| Similar Motifs Found | motif file (matrix) |
| 22 | A C T G A C T G C T G A C T A G A C T G C G T A A C T G | 1e-23 | -5.372e+01 | 33.81% | 22.82% | 56.1bp (68.4bp) | SRSF1(RRM)/Homo\_sapiens-RNCMPT00163-PBM/HughesRNA(0.962) More Information \| Similar Motifs Found | motif file (matrix) |
| 23 | T G C A G T A C A G C T C T G A G T A C C T G A G C T A G A C T G A C T A T G C A G T C A G T C C G T A T C A G | 1e-23 | -5.349e+01 | 2.66% | 0.32% | 50.8bp (69.6bp) | GFY(?)/Promoter/Homer(0.919) More Information \| Similar Motifs Found | motif file (matrix) |
| 24 | A C G T T C A G A G C T C T A G A C T G A C T G G T A C C A G T | 1e-22 | -5.262e+01 | 12.65% | 5.95% | 56.1bp (64.8bp) | PCF/Arabidopsis-Promoters/Homer(0.770) More Information \| Similar Motifs Found | motif file (matrix) |
| 25 | T G A C C G A T A T C G T C G A A G C T T G C A T C G A A T G C G T A C C T A G G T A C G T C A | 1e-21 | -4.947e+01 | 5.91% | 1.84% | 54.1bp (64.9bp) | Unknown5/Drosophila-Promoters/Homer(0.716) More Information \| Similar Motifs Found | motif file (matrix) |
| 26 | G A T C G T C A T C G A A G T C A T G C G T C A C G T A G A T C | 1e-21 | -4.892e+01 | 30.08% | 20.05% | 58.2bp (64.4bp) | MYB96(MYB)/colamp-MYB96-DAP-Seq(GSE60143)/Homer(0.782) More Information \| Similar Motifs Found | motif file (matrix) |
| 27 | A C T G C A G T C G A T T C G A G A C T G T C A T G C A G A T C A T G C | 1e-20 | -4.632e+01 | 12.47% | 6.17% | 58.7bp (67.1bp) | RFX1/MA0365.1/Jaspar(0.717) More Information \| Similar Motifs Found | motif file (matrix) |
| 28 | A T G C T C G A C A G T G C A T C G A T T G A C A G T C C A G T A G T C G T C A | 1e-20 | -4.618e+01 | 10.34% | 4.70% | 53.3bp (64.5bp) | MAC1/MA0326.1/Jaspar(0.738) More Information \| Similar Motifs Found | motif file (matrix) |
| 29 | C G A T A T C G A G C T C T A G A T C G A G C T A G C T G A C T C A G T A T C G C G A T A C T G C A T G A G C T | 1e-19 | -4.584e+01 | 2.66% | 0.41% | 51.6bp (66.1bp) | RUNX2(Runt)/PCa-RUNX2-ChIP-Seq(GSE33889)/Homer(0.716) More Information \| Similar Motifs Found | motif file (matrix) |
| 30 | A C G T C T A G G T C A C A T G A C G T A C T G C G T A A G T C C T G A A T C G C G T A C G T A A C T G C G T A | 1e-19 | -4.479e+01 | 1.06% | 0.02% | 53.9bp (68.1bp) | Pbx3(Homeobox)/GM12878-PBX3-ChIP-Seq(GSE32465)/Homer(0.800) More Information \| Similar Motifs Found | motif file (matrix) |
| 31 | C T G A A G T C C G T A A C T G A G T C C G A T A C T G | 1e-18 | -4.251e+01 | 39.72% | 29.38% | 58.3bp (65.5bp) | MyoG(bHLH)/C2C12-MyoG-ChIP-Seq(GSE36024)/Homer(0.971) More Information \| Similar Motifs Found | motif file (matrix) |
| 32 | G C T A A C G T A C T G A T G C A G C T A G T C C G A T A T C G G A C T A C T G A C T G G A C T C G A T | 1e-18 | -4.204e+01 | 1.42% | 0.09% | 53.6bp (53.5bp) | RUNX1/MA0002.2/Jaspar(0.785) More Information \| Similar Motifs Found | motif file (matrix) |
| 33 | A C T G A T G C C G A T A G C T C G T A C G T A C T G A C A G T C T A G A G T C C G T A C T G A C G T A A C G T | 1e-18 | -4.181e+01 | 0.83% | 0.01% | 54.3bp (12.7bp) | POU2F2/MA0507.1/Jaspar(0.732) More Information \| Similar Motifs Found | motif file (matrix) |
| 34 | A G T C G A T C A T C G A G T C G A C T C A G T G A T C A G T C A G C T A G T C C G A T A G T C | 1e-18 | -4.156e+01 | 4.20% | 1.15% | 53.8bp (68.8bp) | PU.1(ETS)/ThioMac-PU.1-ChIP-Seq(GSE21512)/Homer(0.668) More Information \| Similar Motifs Found | motif file (matrix) |
| 35 | C A G T C A T G T A G C C T A G A C T G A C G T A C G T G C T A | 1e-17 | -4.095e+01 | 23.11% | 14.89% | 57.6bp (67.6bp) | Bgb::run/MA0242.1/Jaspar(0.885) More Information \| Similar Motifs Found | motif file (matrix) |
| 36 | C T G A G A T C A G C T A C G T G A T C T A G C A C G T A C T G A C G T A C T G A C T G A T G C A C G T | 1e-17 | -4.089e+01 | 2.78% | 0.52% | 63.8bp (67.6bp) | Ets1-distal(ETS)/CD4+-PolII-ChIP-Seq(Barski\_et\_al.)/Homer(0.768) More Information \| Similar Motifs Found | motif file (matrix) |
| 37 | G T A C C G A T T A G C T A C G A G T C C T A G T C G A A C G T G T C A G C A T | 1e-17 | -4.001e+01 | 3.49% | 0.84% | 54.2bp (71.9bp) | GFX(?)/Promoter/Homer(0.816) More Information \| Similar Motifs Found | motif file (matrix) |
| 38 | G A C T A G T C T G C A A G T C C T A G C G A T A T C G C T G A A G T C C A G T | 1e-16 | -3.840e+01 | 3.37% | 0.82% | 57.6bp (60.1bp) | TFE3(bHLH)/MEF-TFE3-ChIP-Seq(GSE75757)/Homer(0.939) More Information \| Similar Motifs Found | motif file (matrix) |
| 39 | C T G A A C T G C G T A A C T G A C T G C G T A C T A G T G A C T A G C C G A T A C T G A C T G C T A G | 1e-15 | -3.683e+01 | 4.20% | 1.27% | 56.4bp (59.7bp) | SRSF1(RRM)/Homo\_sapiens-RNCMPT00163-PBM/HughesRNA(0.745) More Information \| Similar Motifs Found | motif file (matrix) |
| 40 | C G T A A T C G C G A T C A T G T G A C G A C T A T C G G C A T T C A G C A G T T A C G T C G A C T A G A C T G | 1e-15 | -3.496e+01 | 2.25% | 0.40% | 49.4bp (62.2bp) | POL009.1\_DCE\_S\_II/Jaspar(0.634) More Information \| Similar Motifs Found | motif file (matrix) |
| 41 | G T C A C A T G G T A C G T C A T A C G G T C A G T C A | 1e-14 | -3.452e+01 | 23.70% | 16.04% | 55.4bp (70.5bp) | NCU08034(RRM)/Neurospora\_crassa-RNCMPT00209-PBM/HughesRNA(0.804) More Information \| Similar Motifs Found | motif file (matrix) |
| 42 | A G T C T C G A C T G A T G C A C T G A T A C G A T C G T C A G T C A G C T G A C T G A T C A G | 1e-14 | -3.451e+01 | 4.02% | 1.24% | 42.9bp (68.0bp) | SPIC/MA0687.1/Jaspar(0.802) More Information \| Similar Motifs Found | motif file (matrix) |
| 43 | T G C A T G A C C T A G C T A G T C G A C T G A C T A G A T C G G A T C A T C G A G C T | 1e-14 | -3.364e+01 | 4.02% | 1.26% | 57.9bp (73.7bp) | REF2(RRM)/Drosophila\_melanogaster-RNCMPT00059-PBM/HughesRNA(0.722) More Information \| Similar Motifs Found | motif file (matrix) |
| 44 | C G A T A G T C C G A T A C G T A G C T C G A T C T G A C G A T G T A C G T C A | 1e-14 | -3.276e+01 | 6.62% | 2.86% | 61.5bp (63.5bp) | KHDRBS1(KH)/Homo\_sapiens-RNCMPT00169-PBM/HughesRNA(0.669) More Information \| Similar Motifs Found | motif file (matrix) |
| 45 | C T A G G C A T C G A T A C T G A G T C A G T C C G T A A G C T C T A G A T C G C G T A C T G A G T C A A G T C | 1e-13 | -3.164e+01 | 1.36% | 0.14% | 45.5bp (65.3bp) | Rfx2(HTH)/LoVo-RFX2-ChIP-Seq(GSE49402)/Homer(0.923) More Information \| Similar Motifs Found | motif file (matrix) |
| 46 | C G T A T C G A C G T A G A C T C T A G A T C G G T A C A C T G C T A G | 1e-13 | -3.096e+01 | 4.79% | 1.79% | 60.1bp (68.1bp) | YY2/MA0748.1/Jaspar(0.905) More Information \| Similar Motifs Found | motif file (matrix) |
| 47 | G C A T C T A G C G T A A G T C T G C A C A T G G C A T G A C T G A C T | 1e-12 | -2.993e+01 | 14.24% | 8.66% | 57.2bp (66.9bp) | Myb/MA0100.2/Jaspar(0.887) More Information \| Similar Motifs Found | motif file (matrix) |
| 48 | A T G C A G T C C G A T A T C G C G T A A C T G A C T G C A T G A C T G A C T G A G T C A C T G A C T G A C T G | 1e-12 | -2.990e+01 | 0.53% | 0.00% | 52.3bp (0.0bp) | PB0076.1\_Sp4\_1/Jaspar(0.771) More Information \| Similar Motifs Found | motif file (matrix) |
| 49 | A G T C A C T G A G T C C G T A C G T A C T G A A G T C A C T G | 1e-12 | -2.943e+01 | 10.70% | 5.95% | 61.4bp (74.5bp) | CG7903(RRM)/Drosophila\_melanogaster-RNCMPT00144-PBM/HughesRNA(0.748) More Information \| Similar Motifs Found | motif file (matrix) |
| 50 | T A G C G C A T A C T G C G T A C A G T T G C A C T G A A T C G T C G A T C G A C T G A T C A G T A G C | 1e-12 | -2.904e+01 | 4.43% | 1.64% | 54.2bp (65.6bp) | PB0022.1\_Gata5\_1/Jaspar(0.760) More Information \| Similar Motifs Found | motif file (matrix) |
| 51 | C T G A C T G A A G T C A T C G A C G T G T A C C T G A | 1e-12 | -2.884e+01 | 8.92% | 4.67% | 56.5bp (68.6bp) | SKO1/Literature(Harbison)/Yeast(0.910) More Information \| Similar Motifs Found | motif file (matrix) |
| 52 | A T G C C G T A A C G T A G T C A G T C A C T G A C T G A C T G | 1e-12 | -2.842e+01 | 3.43% | 1.09% | 62.1bp (68.2bp) | SPDEF/MA0686.1/Jaspar(0.905) More Information \| Similar Motifs Found | motif file (matrix) |
| 53 | C G T A A C G T A C G T A G C T C G T A C G A T C G T A C G T A C G T A A C T G C T A G A G T C C G T A A C G T | 1e-12 | -2.833e+01 | 0.65% | 0.01% | 62.4bp (31.3bp) | dof42(C2C2dof)/col-dof42-DAP-Seq(GSE60143)/Homer(0.662) More Information \| Similar Motifs Found | motif file (matrix) |
| 54 | A C G T T G A C C G A T A T C G A C T G A C T G T A C G A C G T A C T G C T A G A C T G A G T C A G T C A C G T | 1e-12 | -2.833e+01 | 0.65% | 0.01% | 43.9bp (42.4bp) | TCP16(TCP)/colamp-TCP16-DAP-Seq(GSE60143)/Homer(0.689) More Information \| Similar Motifs Found | motif file (matrix) |
| 55 | A C G T T A G C A C G T A C T G C G A T C G A T A T C G C G T A C T G A C G T A A G C T G T C A G T C A C T A G | 1e-12 | -2.833e+01 | 0.65% | 0.01% | 39.4bp (72.7bp) | RAV1(RAV)/colamp-RAV1-DAP-Seq(GSE60143)/Homer(0.653) More Information \| Similar Motifs Found | motif file (matrix) |
| 56 | A T C G A C T G C T G A C G A T C T G A A G T C G A C T C T A G A G T C | 1e-12 | -2.821e+01 | 4.96% | 2.00% | 52.2bp (66.8bp) | SOK2/SOK2\_BUT14/4-SUT1(Harbison)/Yeast(0.629) More Information \| Similar Motifs Found | motif file (matrix) |
| 57 | A G T C T C G A A T C G A C G T A G C T C G A T A G T C C G T A C T A G A C G T A G C T | 1e-12 | -2.819e+01 | 1.71% | 0.29% | 56.0bp (55.4bp) | IRF3(IRF)/BMDM-Irf3-ChIP-Seq(GSE67343)/Homer(0.838) More Information \| Similar Motifs Found | motif file (matrix) |
